# Giant breast lipoma: A case report

**DOI:** 10.1016/j.ijscr.2024.109787

**Published:** 2024-05-22

**Authors:** Fatima E.L. Hassouni, Amal Benchine, Salma Marrakchi, Lamiaa Rouas, Khalid Fathi, Mounia Youssfi

**Affiliations:** a Department of Obstetrics and Gynecology, Oncology and High-Risk Pregnancies, Maternity Hospital Souissi, Ibn Sina University Hospital, Rabat, Morocco; b Mohamed V University, Rabat, Morocco; c Department of Pathology, Ibn Sina University Hospital, Rabat, Morocco; d Department of Radiology, Ibn Sina University Hospital, Rabat, Morocco

**Keywords:** Breast neoplasms, Giant breast lipoma, A case report, Mammography, Liposuction, Surgical excision

## Abstract

**Introduction:**

Lipoma is the most common benign tumor of adipose tissue. Giant lipoma of the breast is defined as lesions larger than 10 cm and weighing more than 1000 g. A breast lipoma rapidly enlarging and fast growing; can be managed as a malignant tumor. It is crucial to make a correct diagnosis to prevent an overtreatment.

**Case presentation:**

A 48-year-old patient presented with a painless, huge rapidly growing tumor in her right breast. Physical examination and imaging studies was suggestive of benign lipomatous breast tumor: A breast lipoma, a fibroadenolipoma or adenolipoma, an angiolipoma, or a breast fatty hamartoma. The patient underwent surgical excision of the mass, and histological examination confirmed the diagnosis of a giant breast lipoma.

**Discussion:**

Giant breast lipoma is a rare benign tumor that develops in the breast tissue. They can mimic various breast conditions, even neoplastic conditions. Giant breast lipomas are often treated with surgical excision to avoid recurrence.

**Conclusion:**

Giant breast lipoma rapidly growing can pose a diagnostic challenge due to its resemblance to various benign or malignant pathologies. Unnecessary invasive investigations can be avoided with better understanding and improved imaging-based diagnosis of giant breast lipoma.

## Highlights

• Giant breast lipomas are relatively rare.
• A breast lipoma rapidly enlarging can be managed as a malignant tumor.
• Unnecessary invasive investigations can be avoided with better understanding and improved imaging-based diagnosis of giant breast lipoma
• Surgical excision remains the main treatment option for giant breast lipoma

## 1 Introduction

Lipomas are the most common benign mesenchymal tumor composed of mature fat cells [1]. They can occur at any age but they most appear between the ages of 40 and 60 years. Although lipomas are common, giant breast lipomas are relatively rare due to their localization and there size [2]. Giant lipoma of the breast is defined as lesions larger than 10 cm and weighing more than 1000 g [1]. A breast lipoma rapidly enlarging and fast growing; can be managed as a malignant tumor. It is crucial to make a correct diagnosis to prevent an overtreatment [2]. We report a case of 48-year-old female who presented with a giant lipoma in the right breast, causing breast asymmetry.

Our work has been reported in line with the SCARE Guidelines 2023 criteria [3].

## 2 Case report

A 48-year-old Arab patient was admitted to our university hospital's gynecology ward with 6 month's history of discomfort due to increasing lump in the right breast. The patient has a 3 years history of systemic arterial hypertension on drug therapy. She did not have a family history of breast carcinoma or breast trauma and she had no history of smoking. Physical examination of the right breast revealed firm, mobile, painless, well defined mass, measuring approximately 10 × 12 cm. There were no nipple discharge or skin changes. The left breast was normal and axillary lymphadenopathy was absent.

The mammographic examination reveals a huge right breast mass located in the upper periareolar region. It appears grossly oval-shaped, well-circumscribed, encapsulated, with regular contours and a radiolucent fatty density that distinguishes it from the rest of breast tissue. There were no associated calcifications. Ultrasonography confirms the oval-shaped and well-circumscribed character of the breast mass. It presents a homogeneous hyperechoic appearance with regular contours, aligned along a major axis parallel to the skin surface. The mass exerts a significant mass effect and measures approximately 10 × 9 cm with breast imaging-reporting and data system (BI-RADS) assessment category 3. However, no signs of malignancy were detected mammographically or sonographically.

**Fig. 1:**
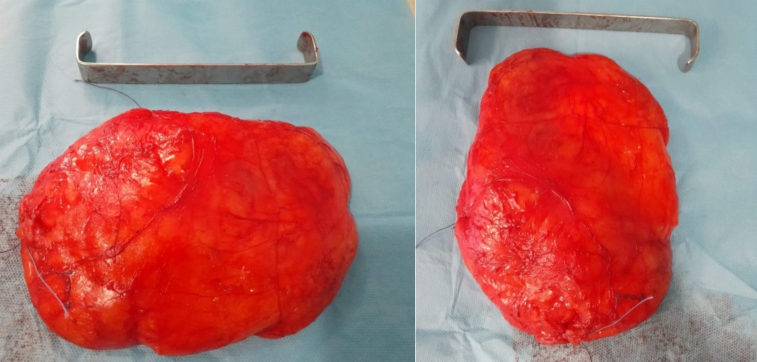
The tumor mass after excision (the size: 14 × 13 × 4 cm).

**Fig. 2:**
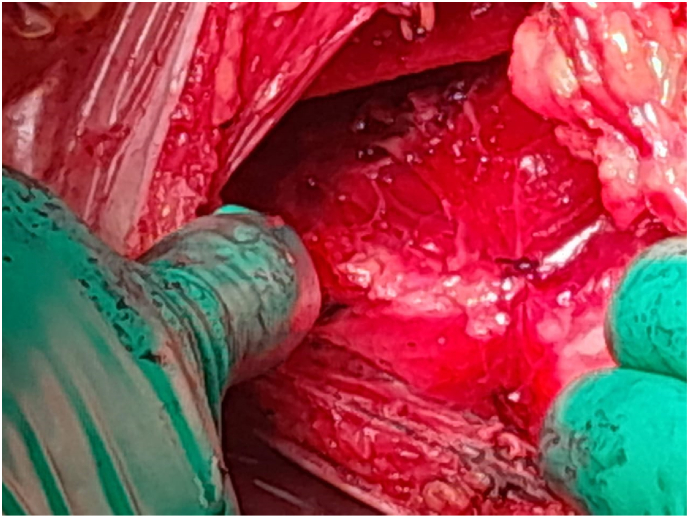
The Intraoperative appearance of the pectoralis major muscle.

**Fig. 3:**
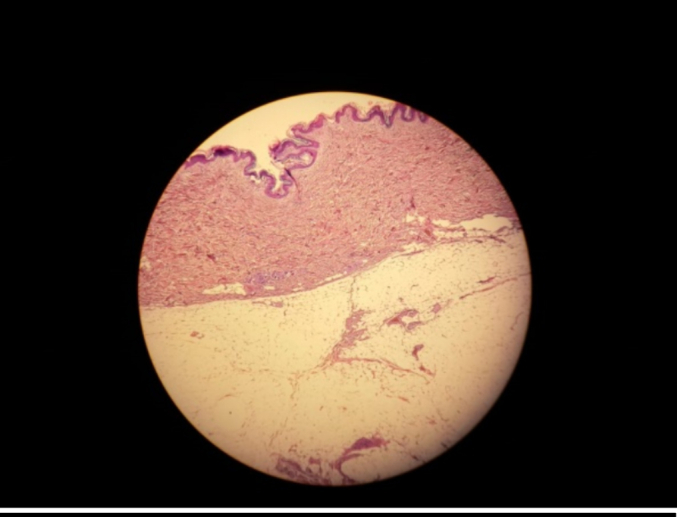
Subcutaneous well circumscribed mature dispose proliferation with thin septa. HE ×4.

**Fig. 4:**
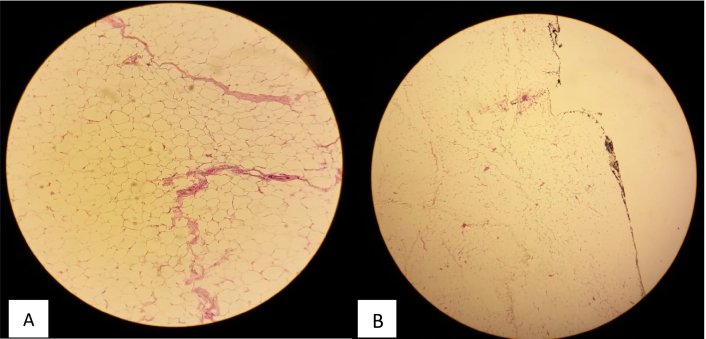
A. Paucicellular fibrous septa separating globules of matures adipocytes. Their nuclei are small compressed nearly invisible. HE ×40. B. Breast lipoma. Pushy positive deep margins. HE ×4.

In view of the large size of the lesion, a surgical excision was performed under general anesthesia. The mass was approached via a lateral incision by a curvilinear lateral incision in order to plan a possible mastopexy in the future. The mass was excised completely (Fig. 1) and the pectoralis major muscle was exposed (Fig. 2). The resected specimen showed a well-demarcated, yellowish, soft nodule, measuring 14 × 13 × 4 cm and weighted 1100 g. Histological analysis of the excised mass showed adipose tissue without evidence of atypia or malignant change and confirmed the diagnosis of lipoma (Fig. 3, Fig. 4).

## 3 Discussion

Lipoma is the most common benign neoplasm originating from adipose tissue, it constitute approximately 16 % of all mesenchymal tumors, with a prevalence rate of 2.3 cases per 1000 individuals [2]. It can occur in any parts of the body, most commonly found in the trunk, upper extremity, legs, and estimated about 20 % in the chest wall [4]. The breast is paradoxically described as a common and uncommon site for this pathology [1] [5].

Giant breast lipoma is a rare benign tumor that develops in the breast tissue. It characterized by a slow-growing mass causing breast enlargement and distortion, and occasionally leads to discomfort or pain. Breast lipoma is classified as a giant lipoma if its size exceeds 10 cm in any one dimension or weighs at least 1000 g [4]. Giant breast lipoma can be difficult to diagnose as they may be confused with other breast masses, such as fibroadenomas, phyllodes tumors, duct papilloma and malignant lump [2]. However, the diagnosis of this type of tumors usually involves clinical examination, imaging studies, and biopsy. Giant lipomas of the breast generally present as well-defined, smooth, and possibly slightly lobulated masses. These tumors are usually soft or semi-firm to the touch and can be mobile when palpated [4]. Fat necrosis of the breast is a benign pathology, however, post-traumatic fat necrosis can have variable manifestations that may sometimes mimic malignancy, depending on its stage of evolution [6].

Ultrasonography (US) and mammography are the first-line examination for routine imaging in the diagnosis of breast diseases [7]. In mammography, lipoma appears as a round or oval radiolucent mass with well-defined thin capsule [8]. When a totally-fatty circumscribed mass is found, an accurate mammographical diagnosis can be made and further investigation is then unnecessary. Occasionally, lipoma may contain typical ring-like calcifications due to fat necrosis [7]. The ultrasound plays an important role in ruling out malignancy and suggesting the diagnosis of lipoma. Breast lipoma appears as oval, homogeneous, lobulated, solid masses with an echogenicity similar to that of normal fat [1]. Magnetic resonance imaging (MRI) is the preferred imaging modality for diagnosing and evaluating a large lipomas, particularly for lesions larger than 5 cm, with rapid growth [9]. Achieving the correct diagnosis might be challenging, as this mass may exhibit atypical features that can mimic other breast tissue masses, necessitating an invasive approach such as Fine Needle Aspiration Cytology (FNAC) or punch biopsy [2].

There are various interventional methods for giant lipoma of the breast, including deoxycholate injection, and liposuction. However, surgical resection is the most effective treatment employed in the management of giant breast lipoma, [2]. The surgical approach may involve traditional excision techniques or liposuction-assisted excision, depending on the size and location of the lipoma [2]. A liposuction technique is more attractive especially in areas where larger scars should be avoided because of the potential for improving cosmetic results. However, it has been shown to increase the risk of recurrence, paraesthesias, edema, numbness and hematoma formation due to incomplete capsule removal [2] [4]. Another challenge for aesthetic surgeons is the potential requirement for breast reconstruction to prevent asymmetry in such cases.

In our case, almost complete right breast parenchyma was replaced by lipoma. Clinical, radiological was suggestive of tumor classified as BI-RADS 3 but the definitive diagnosis was established according to the histopathological examination that was done after the excision of the mass.

Even for patients who have undergone complete removal of these lesions, follow-up assessments are suggested as many cases of recurrences have been reported [1] [10].

## 4 Conclusion

Giant lipoma of breast is very rare. It is often difficult to differentiate it clinically and radiologically from other common breast lesions as this mass may exhibit atypical features. Unnecessary invasive investigations can be avoided with better understanding and improved imaging-based diagnosis of giant breast lipoma, which can be achieved through better knowledge of their typical radiological features, particularly their fatty nature.

## Consent

Consent was obtained from the patient to publish this case report and accompanying images.

## Ethical approval

Ethical approval is not applicable. The case report is not containing any personal information.

## Funding

No funding or grant support.

## Author contribution

Fatima Elhassouni, Mounia Youssfi, Amal Benchine: performed surgery, paper writing and editing.

Mounia Youssfi, Lamiaa rouas, Khalid Fathi: literature review, Supervision.

Fatima Elhassouni, Amal Benchine, salma Marrakchi, Lamiaa Rouas: Manuscript editing, picture editing.

## Guarantor

Fatima Elhassouni.

## Research registration number

Not applicable.

## Conflict of interest statement

The authors declare that they have no competing interests relevant to the content of this article.
